# Net clinical benefit of oral anticoagulants in Asian patients with atrial fibrillation based on a CHA_2_DS_2_-VASc score

**DOI:** 10.1186/s12872-023-03643-8

**Published:** 2023-12-19

**Authors:** Komsing Methavigul, Ply Chichareon, Ahthit Yindeengam, Rungroj Krittayaphong

**Affiliations:** 1 Department of Cardiology, Central Chest Institute of Thailand, 74 Tiwanon road, Nonthaburi, Mueang Nonthaburi 11000 Thailand; 2 Cardiology Unit, Division of Internal Medicine, Faculty of Medicine, Prince of Songkla University, Songkhla, Thailand; 3 Her Majesty Cardiac Center, Faculty of Medicine Siriraj Hospital, Mahidol University, Bangkok, Thailand; 4 Division of Cardiology, Department of Medicine, Faculty of Medicine Siriraj Hospital, Mahidol University, 2 Wanglang Road, Bangkoknoi, Bangkok, 10700 Thailand

**Keywords:** Net clinical benefit, Anticoagulant, Dual antithrombotic, Atrial fibrillation, CHA_2_DS_2_-VASc

## Abstract

**Background:**

This study was conducted to assess the net clinical benefit (NCB) for oral anticoagulant (OAC) in atrial fibrillation (AF) patients according to the CHA_2_DS_2_-VASc score.

**Methods:**

Patients with AF were prospectively recruited in the COOL AF Thailand registry from 2014 to 2017. The incidence rate of thromboembolic (TE) events and major bleeding (MB) was calculated. Cox proportional hazards model was used to compare the TE and MB rate in patients with and without OACs in CHA_2_DS_2_-VASc score of 0–1 and ≥ 2, respectively. The survival analysis was performed based on CHA_2_DS_2_-VASc score. The NCB of OACs was defined as the TE rate prevented minus the MB rate increased multiplied by a weighting factor.

**Results:**

A total of 3,402 AF patients were recruited. An average age of patients was 67.38 ± 11.27 years. Compared to non-anticoagulated patients, the Kaplan Meier curve showed anticoagulated patients with CHA_2_DS_2_-VASc score of 2 or more had the lower thromboembolic events with statistical significance (*p* = 0.043) and the higher MB events with statistical significance (*p* = 0.018). In overall AF patients, there were positive NCB in warfarin patients with CHA_2_DS_2_-VASc score of 3 or more while there were positive NCB in DOACs patients regardless of CHA_2_DS_2_-VASc score. Females with CHA_2_DS_2_-VASc score of 3 or more had a positive NCB regardless of OACs type. Good anticoagulation control (TTR ≥65%) improved an NCB in males with CHA_2_DS_2_-VASc score of 3 or more.

**Conclusions:**

AF patients with CHA_2_DS_2_-VASc score of 3 or more regardless warfarin or DOACs had a positive NCB. The NCB of OACs was more positive for DOACs compared to warfarin and for females compared to males.

## Background

Ischemic stroke prevention is of paramount importance in patients with atrial fibrillation (AF). Previous clinical trials have shown that oral anticoagulants (OACs) can be used for prevention of ischemic stroke in patients with AF [1–3]. CHA_2_DS_2_-VASc score is recommended for selection of those patients who have a benefit of OACs by all international standard guidelines [4–7].

However, OACs increase the risk of bleeding even in patients with CHA_2_DS_2_-VASc score of 0. The benefit of stroke prevention would be offset by the occurrence of bleeding [8]. Warfarin-associated intracranial hemorrhage (ICH), the most catastrophic complication, is responsible for mortality in 90% of patients treated with warfarin [9]. Several studies have shown the lower risk of ICH in patients treated with direct oral anticoagulants (DOACs) compared with warfarin [10–13].

Several studies have shown that warfarin had a positive net clinical benefit (NCB) in AF patients with high stroke risk [14, 15]. Additionally, DOACs have been studied in elderly with AF and shown a positive NCB as well [16]. The NCB assessment in previous clinical trials comprised ischemic stroke and ICH, however, the extracranial bleeding was not considered. Nevertheless, the AnTicoagulation and Risk Factors In Atrial Fibrillation (ATRIA) study has demonstrated that major extracranial bleeding is still detrimental and causing many hospital admission in anticoagulated AF patients [9].

Until now, there was a lack of data on the NCB between thromboembolic and major bleeding events in patients treated with warfarin or DOACs. This study was conducted to assess the NCB of OACs in AF patients according to their CHA_2_DS_2_-VASc score.

## Methods

### Study population and setting

Patients with AF aged 18 years or more in 27 hospitals including university and/or general hospitals in Thailand were prospectively recruited from 2014 to 2017 to the COhort of antithrombotic use and Optimal INR Level in patients with non-valvular atrial fibrillation in Thailand (COOL AF Thailand) study [17]. Patients with prosthetic heart valve, rheumatic mitral valve disease, recent ischemic stroke within 3 months, transient reversible cause of AF, life expectancy below 3 years, pregnancy, thrombocytopenia (< 100,000/mm^3^), myeloproliferative diseases, refusal to be enrolled, and/or could not visit for follow-up were excluded.

The trial protocol was approved by the Central Research Ethics Committee (CREC). Written informed consent was obtained by all participated patients. The study was conducted in compliance with the Declaration of Helsinki and the International Conference on Harmonization for Good Clinical Practice Guidelines (ICH-GCP).

### Data collection and outcomes

Baseline demographic and clinical data of patients with AF were collected and recorded. Patients were follow-up every 6 months until 3 years. Each patient data was recorded on electronic case record form via web-based system. The following clinical event data during follow-up visit were recorded: thromboembolic events including ischemic stroke, transient ischemic attack (TIA) and/or systemic embolization and major bleeding including ICH and/or extracranial bleeding.

Ischemic stroke was defined as a sudden onset of neurological deficit that lasted at least 24 h without ICH by computed tomography (CT) or magnetic resonance imaging (MRI). TIA was defined as a sudden neurological deficit that lasted less than 24 h. Systemic embolism was defined as the disruption of blood flow to other arteries such as acute limb arterial occlusion or acute mesenteric arterial occlusion.

Major bleeding was defined as fatal bleeding, critical organ bleeding including ICH, intraspinal, intraocular/retinal, retroperitoneal, intraarticular, pericardial, intramuscular with/without compartment syndrome and/or bleeding causing a fall in hemoglobin level of 2 g/dL or more or leading to 2 or more units of blood transfusion [18].

Each component of the CHA_2_DS_2_ -VASc score was evaluated and recorded as C = congestive heart failure (1 point); H = hypertension (1 point); A = age ≥ 75 years (2 points); D = diabetes mellitus (1 point); S = stroke and/or TIA (2 points); V = vascular disease (1 point); A = age 65–74 years (1 point); and Sc = female sex category (1 point) [19].

### Statistical analysis

Categorical data are presented as percentage and number. Continuous data are presented as mean ± standard deviation (SD). The annual incidence rate of thromboembolic and major bleeding events in patients within each group of the CHA_2_DS_2_-VASc score was shown as rate per 100 person-years. Univariable Cox proportional hazards model was used to compare the annual incidence rate of both events in patients with and without OACs in CHA_2_DS_2_-VASc score of 0–1 and ≥ 2, respectively. Hazard ratio (HR) was adjusted by symptoms and pattern of AF, cardiovascular implantable electronic devices (CIEDs), dyslipidemia, chronic kidney disease (CKD), dementia, a history of bleeding, alcohol use, left ventricular ejection fraction (LVEF) < 50%, antiplatelet drugs. The results were illustrated with adjusted HR and 95% confidence interval (CI). The survival analysis from the outcome of interest in each group of the CHA_2_DS_2_-VASc score are presented with Kaplan-Meier curve. A *p*-value < 0.05 was considered as statistical significance.

The NCB of OACs was defined as the annual incidence rate of thromboembolic events (TE rate) prevented minus the annual incidence rate of major bleeding events (MB rate) increased multiplied by a weighting factor (WF) [14]. The following equation was demonstrated below:

NCB = (TE rate _no OACs_ – TE rate _on OACs_) – [WF x (MB rate _on OACs_ – MB rate _no OACs_)]

The WF reflected the relative impact of death and/or disability of MB rate in patients with warfarin or DOACs versus suffering from TE rate in those with no OACs. We assigned a WF of 1.0 as a base case and also provided additional sensitivity analyses by using WF of 1.5 and 2.0.

The NCB of warfarin and DOACs was calculated in overall, male and female AF patients according to the NCB of OACs in warfarin and DOACs cohort, respectively.

## Results

A total of 3,402 AF patients were recruited in the COOL AF Thailand study. The mean age of patients was 67.38 ± 11.27 years (Table 1). Patients with OACs at baseline were older than those without OACs. Nearly 60% of patients were male sex. Most patients had hypertension and about one-third had a history of heart failure or left ventricular systolic dysfunction. Most patients in this study had high stroke risk (CHA_2_DS_2_-VASc score of 2 or more) and low bleeding risk (HAS-BLED less than 3). About one-fifth of patients had concomitant antiplatelet therapy. The proportion of patients treated with antiplatelets in the group without OACs was higher than those with OAC. Warfarin was commonly used in this study. The average time in therapeutic range (TTR) in patients with warfarin was 53.6 ± 26.4%.

**Table 1:** Baseline characteristics of atrial fibrillation patients

Baseline demographic data	Total patients (n = 3402)	Patients with OACs (n = 2566)	Patients without OACs (n = 836)	*p*-value
Age (years)	67.38 ± 11.27	68.37 ± 10.70	64.32 ± 12.39	< 0.001*
Male gender	1980 (58.2%)	1452 (56.6%)	528 (63.2%)	0.001*
Symptomatic AF	2618 (77.0%)	77.0 (77.0%)	645 (77.2%)	0.875
Atrial fibrillation				< 0.001*
- Paroxysmal	1148 (33.7%)	778 (30.3%)	370 (44.3%)	
- Persistent	643 (18.9%)	481 (18.7%)	162 (19.4%)	
- Permanent	1611 (47.4%)	1307 (50.9%)	304 (36.4%)	
CIED	341 (10.0%)	270 (10.5%)	71 (8.5%)	0.090
Smoking	678 (19.9%)	473 (18.4%)	205 (24.5%)	< 0.001*
Dyslipidemia	1915 (56.3%)	1506 (58.7%)	409 (48.9%)	< 0.001*
CKD	1754 (51.6%)	1390 (54.2%)	364 (43.5%)	< 0.001*
Dementia	29 (0.9%)	25 (1.0%)	4 (0.5%)	0.176
History of bleeding	323 (9.5%)	272 (10.6%)	51 (6.1%)	< 0.001*
- Major bleeding	70 (2.1%)	54 (2.1%)	16 (1.9%)	0.736
- Minor bleeding	253 (7.4%)	218 (8.5%)	35 (4.2%)	***< 0.001***
- ICH	31 (0.9%)	19 (0.7%)	12 (1.4%)	0.066
History of thromboembolic event	604 (17.8%)	550 (21.4%)	54 (6.5%)	***< 0.001***
- Ischemic stroke	485 (14.3%)	445 (17.3%)	40 (4.8%)	***< 0.001***
- TIA	126 (3.7%)	111 (4.3%)	15 (1.8%)	***0.001***
- Systemic embolism	25 (0.7%)	25 (1.0%)	0 (0.0%)	***0.004***
Alcohol use	140 (4.1%)	86 (3.4%)	54 (6.5%)	< 0.001*
LVEF (%)	59.87 ± 14.71	59.25 ± 15.03	61.76 ± 13.51	< 0.001*
TTR (%)	53.6 ± 26.4	53.6 ± 26.4	-	-
TTR < 65%	1432 (64.1%)	1432 (64.1%)	-	-
TTR 65 to < 70%	168 (7.5%)	168 (7.5%)	-	-
TTR ≥ 70%	633 (28.3%)	633 (28.3%)	-	-
**Components of** **CHA**_**2**_ **DS**_**2**_ **-VASc score**				
- History of heart failure/LV systolic dysfunction	1045 (30.7%)	810 (31.6%)	235 (28.1%)	0.060
- Hypertension	2328 (68.4%)	1861 (72.5%)	467 (55.9%)	< 0.001*
- Age ≥ 75 years	979 (28.8%)	799 (31.1%)	180 (21.5%)	< 0.001*
- Diabetes mellitus	839 (24.7%)	690 (26.9%)	149 (17.8%)	< 0.001*
- Previous stroke or TIA	592 (17.4%)	538 (21.0%)	54 (6.5%)	< 0.001*
- Vascular disease	581 (17.1%)	441 (17.2%)	140 (16.7%)	0.769
- Age 65–74 years	1094 (32.2%)	887 (34.6%)	207 (24.8%)	< 0.001*
- Female sex	1422 (41.8%)	1114 (43.4%)	308 (36.8%)	0.001*
**CHA**_**2**_ **DS**_**2**_ **-VASc score**				< 0.001*
− 0	196 (5.8%)	61 (2.4%)	135 (16.1%)	
− 1	422 (12.4%)	236 (9.2%)	186 (22.2%)	
- ≥ 2	2784 (81.8%)	2269 (88.4%)	515 (61.6%)	
**HAS-BLED score**				0.482
− 0–2	2863 (84.2%)	2153 (83.9%)	710 (84.9%)	
- ≥ 3	539 (15.8%)	413 (16.1%)	126 (15.1%)	
**Antithrombotic medications**				
Antiplatelets	890 (26.2%)	308 (12.0%)	582 (69.6%)	< 0.001*
- Aspirin	784 (23.0%)	263 (10.2%)	521 (62.3%)	< 0.001*
- P2Y_12_ inhibitors	200 (5.9%)	81 (3.2%)	119 (14.2%)	< 0.001*
Oral anticoagulants				
- Warfarin	2338 (68.7%)	2338 (91.1%)	0 (0.00%)	< 0.001*
- Direct thrombin inhibitor	82 (2.4%)	82 (3.2%)	0 (0.00%)	< 0.001*
- Factor Xa inhibitors	145 (4.3%)	145 (5.7%)	0 (0.00%)	< 0.001*

Data were displayed as mean ± SD or n (%), n = numbers, SD = standard deviation, AF = atrial fibrillation, OACs = oral anticoagulants, CIED = cardiovascular implantable electronic device, CKD = chronic kidney disease, ICH = intracranial hemorrhage, GI = gastrointestinal, LVEF = left ventricular ejection fraction, TTR = time in therapeutic range, LV = left ventricular, TIA = transient ischemic attack

### Risk of thromboembolic events according to the CHA_2_DS_2_-VASc score

**Fig. 1:**
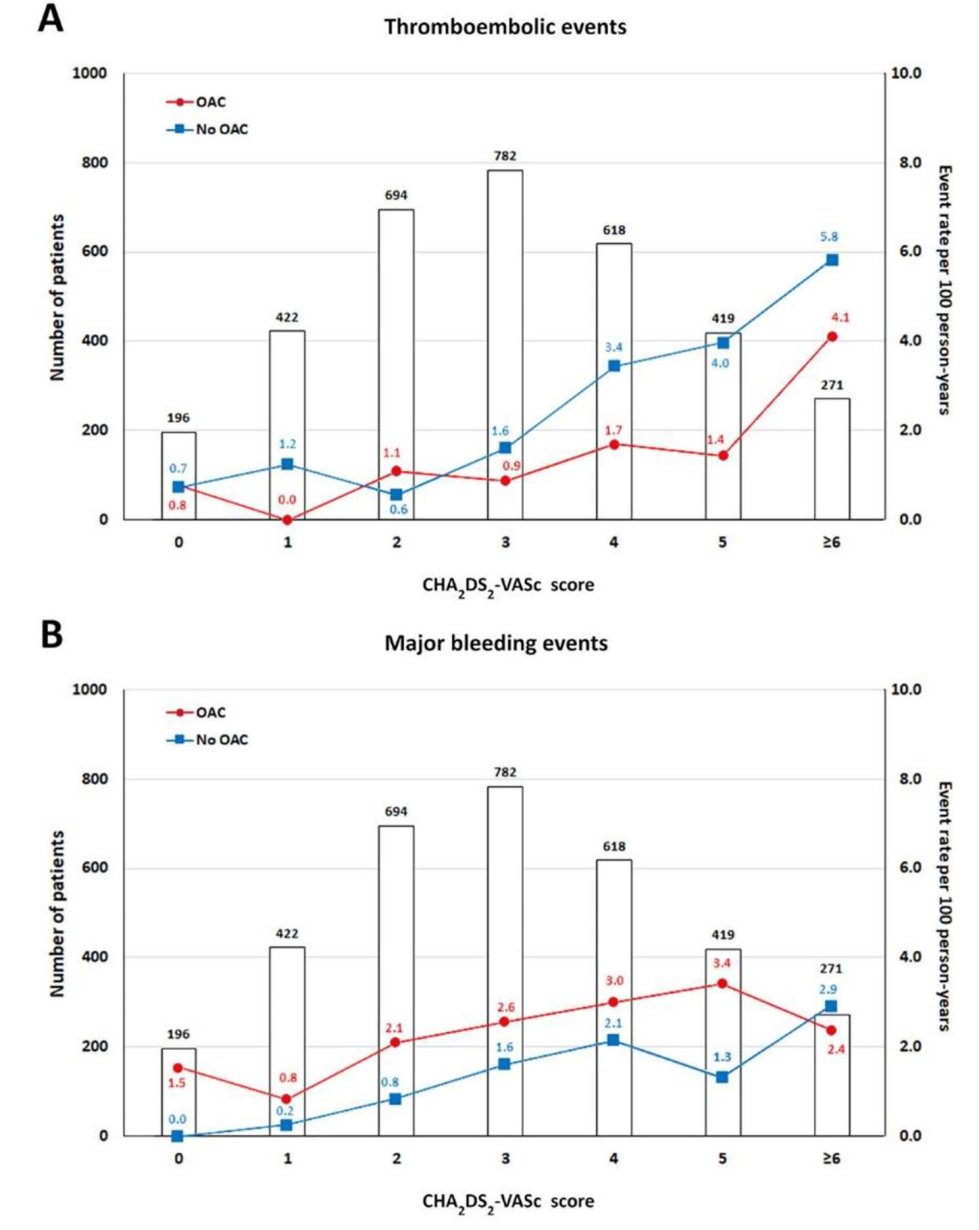
Cumulative incidences in AF patients with or without OACs based on CHA_2_DS_2_-VASc score (**A**) thromboembolic events (**B**) major bleeding events

Annual thromboembolic (TE) events of AF patients with or without OACs increased according to CHA_2_DS_2_-VASc score (Table 2). The cumulative incidences of thromboembolic events increased following CHA_2_DS_2_-VASc score as well (Fig. 1).

**Table 2:** Annual thromboembolic events in atrial fibrillation patients with or without oral anticoagulants (OACs) stratified by CHA_2_DS_2_-VASc score (CI = confidence interval)

CHA_2_DS_2_-VASc score	Number of patients	Number of events	100 person-years	Rate per 100 person-years	95% CI
All patients					
0	196	3	4.0	0.74	0.15–2.19
1	422	5	8.9	0.56	0.18–1.31
2	694	14	14.5	0.96	0.53–1.62
3	782	17	16.7	1.02	0.59–1.63
4	618	26	13.0	2.01	1.31–2.93
5	419	17	9.1	1.86	1.09–2.99
≥ 6	271	25	5.6	4.43	2.89–6.59
Total	3402	107	71.9	1.49	1.22–1.80
OACs					
0	61	1	1.3	0.77	0.02–4.29
1	236	0	4.9	0.00	-
2	539	12	11.0	1.09	0.56–1.91
3	641	12	13.6	0.88	0.46–1.54
4	515	18	10.6	1.69	1.00-2.68
5	352	11	7.6	1.44	0.72–2.59
≥ 6	222	19	4.6	4.11	2.49–6.459
Total	2566	73	53.6	1.36	1.07–1.71
No OACs					
0	135	2	2.7	0.73	0.09–2.68
1	186	5	4.0	1.24	0.41–2.92
2	155	2	3.5	0.56	0.07–2.06
3	141	5	3.1	1.61	0.52–3.76
4	103	8	2.3	3.44	1.50–6.85
5	67	6	1.5	3.97	1.47–8.71
≥ 6	49	6	1.0	5.83	2.20-13.06
Total	836	34	18.3	1.86	1.29–2.60

The overall TE rate in non-anticoagulated AF patients with CHA_2_DS_2_-VASc score of 2 or more was 2.34% (95%CI 1.55–3.42%) while it was 0.73% (95%CI 0.09–2.68%) and 1.24% (95%CI 0.41–2.92%) in those with score of 0 or 1, respectively.

In the group with CHA_2_DS_2_-VASC score of 0–1, the rate of TE in patients treated with OAC was similar to those without OACs (0.16 vs. 1.03, p value 0.100). In the group with CHA_2_DS_2_-VASC score of 2 or more, the rate of TE in patients with OACs was numerically lower than those without OACs (1.52 vs. 2.34, p value 0.740), however, the difference was not statistically significant (adjusted HR 0.74, 95%CI 0.41–1.34, p value 0.74) (Table 3). In warfarin cohort, AF patients without OACs had increased TE rate in those patients with CHA_2_DS_2_-VASc score of 2 or more compared with those with score of 0–1 as well as any OACs cohort. However, there was a trend in reduced TE rate in those patients with CHA_2_DS_2_-VASc score of 2 or more with non-statistical significance (adjusted HR 0.77; 95%CI 0.43 to 1.40; *p* = 0.391) (Table 3). In DOACs cohort, AF patients without OACs had incremental TE rate in those patients with CHA_2_DS_2_-VASc score of 2 or more compared with those with score of 0–1 as well as aforementioned cohort. As warfarin cohort, DOACs could reduce TE rate in those patients with CHA_2_DS_2_-VASc score of 2 or more with no statistical significance (adjusted HR 0.56; 95%CI 0.13 to 2.48; *p* = 0.444) (Table 3).

**Table 3:** Risk of thromboembolic events of atrial fibrillation patients based on CHA_2_DS_2_-VASc score and oral anticoagulants (OACs)

Antithrombotic strategy	Thromboembolic events		
	Annual incidence rate	Adjusted HR (95%CI)	*P* value
**Any OACs cohort**			
CHA_2_DS_2_-VASc of 0–1			
- OACs	0.16 (0.01–0.90)	0.13 (0.01–1.49)	0.100
- No OACs	1.03 (0.41–2.12)	Reference	
CHA_2_DS_2_-VASc of 2 or more			
- OACs	1.52 (1.19–1.91)	0.74 (0.41–1.34)	0.740
- No OACs	2.34 (1.55–3.42)	Reference	
**Warfarin cohort**			
CHA_2_DS_2_-VASc of 0–1			
- Warfarin	0.19 (0.01–1.07)	0.13 (0.01–1.57)	0.108
- No OACs	1.03 (0.41–2.12)	Reference	
CHA_2_DS_2_-VASc of 2 or more			
- Warfarin	1.58 (1.23-2.00)	0.77 (0.43–1.40)	0.391
- No OACs	2.34 (1.55–3.42)	Reference	
**DOACs cohort**			
CHA_2_DS_2_-VASc of 0–1			
- DOACs	-	-	-
- No OACs	1.03 (0.41–2.12)	Reference	
CHA_2_DS_2_-VASc of 2 or more			
- DOACs	0.8 (0.17–2.37)	0.56 (0.13–2.48)	0.444
- No OACs	2.34 (1.55–3.42)	Reference	

AF = atrial fibrillation, HR = hazard ratio, 95%CI = 95% confidence interval, OACs = oral anticoagulants, DOACs = direct oral anticoagulants

Variables for adjusted: Symptoms and pattern of AF, CIEDs, dyslipidemia, CKD, dementia, a history of bleeding, alcohol use, LVEF < 50%, antiplatelet drugs

**Fig. 2:**
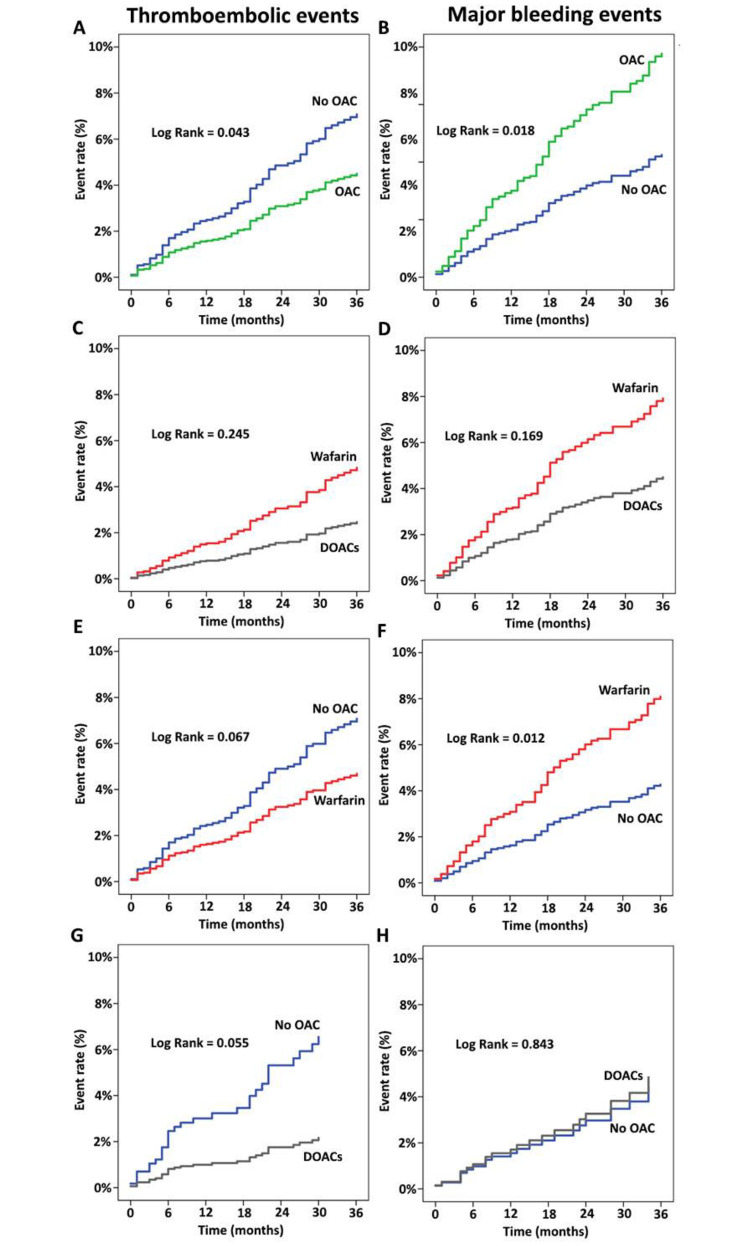
Kaplan Meier curve of thromboembolic events and major bleeding events in AF patients with CHA_2_DS_2_-VASc score ≥ 2 between OACs and no OACs (**A** and **B**), DOACs and warfarin (**C** and **D**), warfarin and no OACs (**E** and **F**), DOACs and no OACs (**G** and **H**)

The Kaplan Meier curve showed anticoagulated AF patients with CHA_2_DS_2_-VASc score of 2 or more had the lower TE rate compared to those non-anticoagulated patients with statistical significance (*p* = 0.043) (Fig. 2A) while there was comparable TE rate in AF patients taking DOACs and warfarin (*p* = 0.245) (Fig. 2C). Nevertheless, there were trends in lower TE rate in AF patients taking warfarin (*p* = 0.067) and DOACs (*p* = 0.055) compared to no OACs (Fig. 2E and G).

### Risk of major bleeding events based on CHA_2_DS_2_-VASc score

Annual MB events of overall AF patients with or without OACs increased according to CHA_2_DS_2_-VASc score (Table 4). The cumulative incidences of MB events increased following CHA_2_DS_2_-VASc score as well (Fig. 1).

**Table 4:** Annual major bleeding events in atrial fibrillation patients with or without oral anticoagulants (OACs) stratified by CHA_2_DS_2_-VASc score (CI = confidence interval)

CHA_2_DS_2_-VASc score	Number of patients	Number of events	100 person-years	Rate per 100 person-years	95% CI
All patients					
0	196	2	4.0	0.49	0.06–1.81
1	422	5	8.9	0.56	0.18–1.31
2	694	26	14.5	1.79	1.17–2.63
3	782	40	16.7	2.39	1.71–3.26
4	618	37	13.0	2.85	2.00-3.92
5	419	28	9.1	3.07	2.04–4.45
≥ 6	271	14	5.6	2.48	1.37–4.19
Total	3402	152	71.9	2.11	1.79–2.48
OACs					
0	61	2	1.3	1.54	0.19–5.56
1	236	4	4.9	0.82	0.22–2.09
2	539	23	11.0	2.10	1.33–3.14
3	641	35	13.6	2.57	1.79–3.58
4	515	32	10.6	3.01	2.06–4.26
5	352	26	7.6	3.41	2.23–5.01
≥ 6	222	11	4.6	2.38	1.19–4.28
Total	2566	133	53.6	2.48	2.08–2.94
No OACs					
0	135	0	2.7	0.00	-
1	186	1	4.0	0.25	0.01–1.39
2	155	3	3.5	0.85	0.18–2.50
3	141	5	3.1	1.61	0.52–3.76
4	103	5	2.3	2.15	0.71–5.07
5	67	2	1.5	1.32	0.16–4.82
≥ 6	49	3	1.0	2.91	0.62–8.77
Total	836	19	18.3	1.04	0.63–1.62

In the group with CHA_2_DS_2_-VASC score of 2 or more, the incidence of MB events in patients treated with OACs was higher than those without OACs (*p* = 0.018) (Fig. 2B). while there were comparable MB events in AF patients taking DOACs compared to warfarin (*p* = 0.169) (Fig. 2D) and those patients taking DOACs compared to no OACs (*p* = 0.843) (Fig. 2H). Nevertheless, there were higher MB events in AF patients taking warfarin compared to no OACs (*p* = 0.012) (Fig. 2F).

In the Cox regression model, OACs was associated with higher risk of major bleeding than those without OACs (OACs vs. no OAC; adjusted HR 2.29, 95%CI 1.26–4.14, *p* = 0.006). The difference was driven by a significant higher bleeding risk in those who were treated with warfarin (warfarin vs. no OACs; adjusted HR 2.38, 95%CI 1.32–4.32, *p* = 0.004). Compared with no OACs, the risk of major bleeding in those treated with DOACs was numerically higher (DOACs vs. no OACs; adjusted HR 3.62, 95%CI 0.88–14.80), the difference was not statistically significant (*p* = 0.074) (Table 5).

**Table 5:** Risk of major bleeding events of anticoagulant AF patients based on CHA_2_DS_2_-VASc score

Antithrombotic strategy	Major bleeding events		
	Annual incidence rate	Adjusted HR (95%CI)	*P* value
**Any OACs cohort**			
CHA_2_DS_2_-VASc of 0–1			
- OACs	0.97 (0.36–2.11)	11.37 (0.95-135.37)	0.054
- No OACs	0.15 (0.01–0.82)	Reference	
CHA_2_DS_2_-VASc of 2 or more			
- OACs	2.68 (2.23–3.18)	2.29 (1.26–4.14)	0.006*
- No OACs	1.56 (0.93–2.47)	Reference	
**Warfarin cohort**			
CHA_2_DS_2_-VASc of 0–1			
- Warfarin	1.16 (0.42–2.51)	13.69 (1.18–159.40)	0.037*
- No OACs	0.15 (0.01–0.82)	Reference	
CHA_2_DS_2_-VASc of 2 or more			
- Warfarin	2.77 (2.30–3.31)	2.38 (1.32–4.32)	0.004*
- No OACs	1.56 (0.93–2.47)	Reference	
**DOACs cohort**			
CHA_2_DS_2_-VASc of 0–1			
- DOACs	-	-	-
- No OACs	0.15 (0.01–0.82)	Reference	
CHA_2_DS_2_-VASc of 2 or more			
- DOACs	1.60 (0.60–3.53)	3.62 (0.88–14.80)	0.074
- No OACs	1.56 (0.93–2.47)	Reference	

AF = atrial fibrillation, HR = hazard ratio, 95%CI = 95% confidence interval, OACs = oral anticoagulants, DOACs = direct oral anticoagulants

Variables for adjusted: Symptoms and pattern of AF, CIEDs, dyslipidemia, CKD, dementia, a history of bleeding, alcohol use, LVEF < 50%, antiplatelet drugs

*A p value < 0.05 indicates statistical significance

### Net clinical benefit between TE and MB rate based on CHA_2_DS_2_-VASc score

**Fig. 3:**
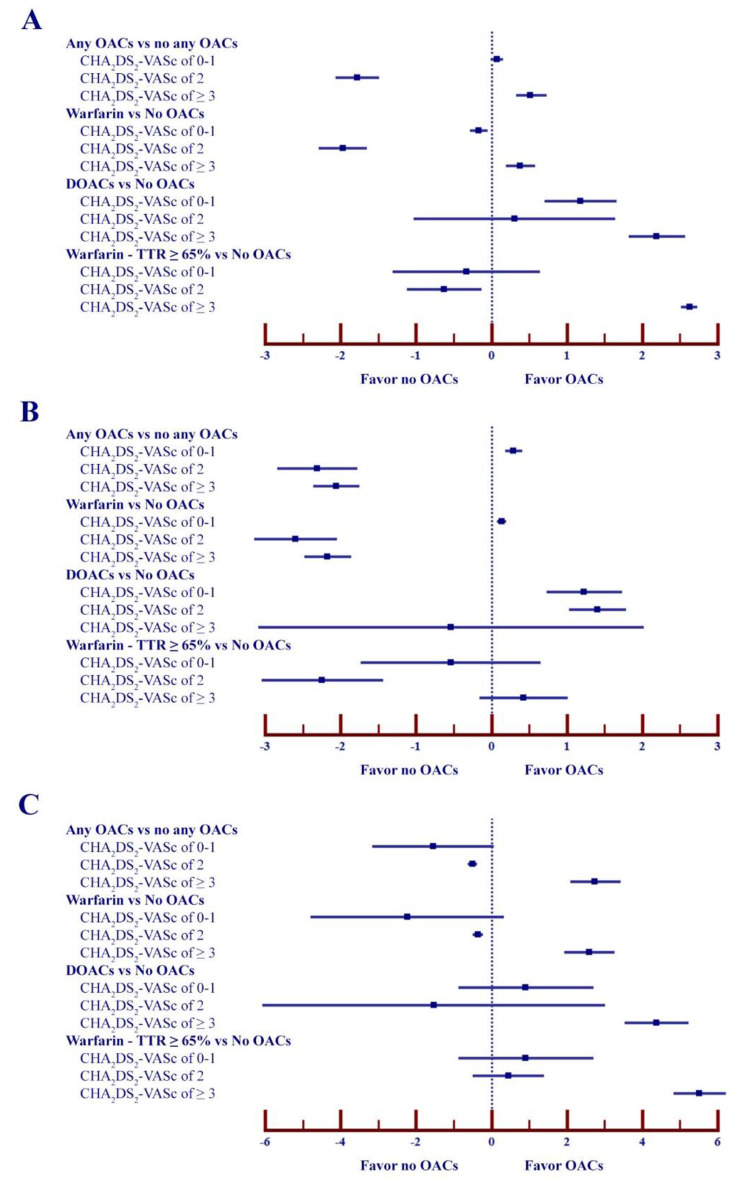
Net clinical benefit of all patients (**A**), male patients (**B**) and female patients (**C**) with atrial fibrillation taking oral anticoagulants based on CHA_2_DS_2_-VASc score

The NCB for any OACs was superior to no OACs in the group with CHA_2_DS_2_-VASc score of 3 or more (NCB 0.52; 95%CI 0.33 to 0.73). The NCB for any OACs was slightly superior to no OACS in patients with CHA_2_DS_2_-VASc score of 0–1 (NCB 0.04; 95%CI 0.04 to 0.05) while inferior in those with CHA_2_DS_2_-VASc score of 2 (NCB − 1.78; 95%CI -2.07 to -1.49). However, the NCB was lower when we assigned WF of 1.5 and 2.0, respectively (Table 6; Fig. 3).

**Table 6:** Net clinical benefit of all patients with atrial fibrillation taking oral anticoagulants based on CHA_2_DS_2_-VASc score

Antithrombotic strategy	NCB (overall) (WF of 1.0)	NCB (overall) (WF of 1.5)	NCB (overall) (WF of 2.0)
Any OACs vs. No OACs			
CHA_2_DS_2_-VASc of 0–1	0.04 (0.04 to 0.05)	-0.37 (-0.79 to 0.05)	-0.78 (-1.62 to 0.05)
CHA_2_DS_2_-VASc of 2	-1.78 (-2.07 to -1.49)	-2.41 (-3.34 to -1.48)	-3.04 (-4.61 to -1.46)
CHA_2_DS_2_-VASc of ≥ 3	0.52 (0.33 to 0.73)	0.04 (-0.31 to 0.39)	-0.44 (-1.34 to 0.46)
Warfarin vs. No OACs			
CHA_2_DS_2_-VASc of 0–1	-0.17 (-0.28 to -0.05)	-0.67 (-1.27 to -0.07)	-1.18 (-2.26 to -0.09)
CHA_2_DS_2_-VASc of 2	-1.97 (-2.29 to -1.65)	-2.69 (-3.68 to -1.70)	-3.41 (-5.07 to -1.75)
CHA_2_DS_2_-VASc of ≥ 3	0.38 (0.19 to 0.58)	-0.13 (-0.49 to 0.23)	-0.65 (-1.57 to 0.27)
DOACs vs. No OACs			
CHA_2_DS_2_-VASc of 0–1	1.18 (0.70 to 1.66)	1.25 (0.92 to 1.59)	1.33 (1.14 to 1.52)
CHA_2_DS_2_-VASc of 2	0.31 (-1.03 to 1.64)	0.73 (-0.13 to 1.59)	1.15 (0.77 to 1.54)
CHA_2_DS_2_-VASc of ≥ 3	2.19 (1.82 to 2.57)	2.08 (0.73 to 3.42)	1.96 (-0.36 to 4.27)
Warfarin (TTR ≥ 65%) vs. No OACs			
CHA_2_DS_2_-VASc of 0–1	-0.33 (-1.31 to 0.64)	-1.02 (-2.86 to 0.82)	-1.70 (-4.41 to 1.01)
CHA_2_DS_2_-VASc of 2	-0.63 (-1.12 to -0.13)	-0.97 (-2.24 to 0.31)	-1.31 (-3.36 to 0.75)
CHA_2_DS_2_-VASc of ≥ 3	2.63 (2.52 to 2.73)	2.72 (2.22 to 3.23)	2.82 (1.72 to 3.93)
DOACs versus Warfarin			
CHA_2_DS_2_-VASc of 0–1	1.35 (0.80 to 1.90)	1.93 (0.92 to 2.93)	2.50 (1.03 to 3.97)
CHA_2_DS_2_-VASc of 2	2.28 (0.96 to 3.60)	3.42 (2.57 to 4.28)	4.56 (4.18 to 4.95)
CHA_2_DS_2_-VASc of ≥ 3	1.81 (1.10 to 2.53)	2.21 (0.60 to 3.82)	2.60 (0.10 to 5.11)
DOACs versus Warfarin (TTR ≥ 65%)			
CHA_2_DS_2_-VASc of 0–1	1.51 (-0.20 to 3.23)	2.27 (-0.30 to 4.84)	3.03 (-0.40 to 6.46)
CHA_2_DS_2_-VASc of 2	0.93 (-0.12 to 1.98)	1.69 (1.25 to 2.14)	2.46 (2.29 to 2.63)
CHA_2_DS_2_-VASc of ≥ 3	-0.43 (-1.19 to 0.33)	-0.65 (-2.34 to 1.04)	-0.87 (-3.48 to 1.75)

NCB = net clinical benefit, WF = weighting factor, OACs = oral anticoagulants, DOACs = direct oral anticoagulants, TTR = time in therapeutic range

In the analysis of warfarin versus no OACs, there were positive NCB for warfarin in patients with CHA_2_DS_2_-VASc score of 3 or more (NCB 0.38; 95%CI 0.19 to 0.58) while there were negative NCB in patients with CHA_2_DS_2_-VASc score of 0–1 (NCB − 0.17; 95%CI

− 0.28 to − 0.05) and those with CHA_2_DS_2_-VASc score of 2 (NCB − 1.97; 95%CI -2.29 to -1.65). However, the NCB was lower when we assigned WF of 1.5 and 2.0, respectively. In the analysis of only well-controlled warfarin (TTR ≥ 65%) compared to no OACs, there was more positive NCB in patients with CHA_2_DS_2_-VASc score of 3 or more (NCB 2.63; 95%CI 2.52 to 2.73). In addition, there has been still positive NCB in those patients despite we assigned WF of 1.5 and 2.0, respectively (Table 6; Fig. 3).

The NCB for DOACs was higher than no OACs in patients with CHA_2_DS_2_-VASc score of 0–1 (NCB 1.18; 95%CI 0.70 to 1.66) and those with CHA_2_DS_2_-VASc score of 3 or more (NCB 2.19; 95%CI 1.82 to 2.57). There were neutral NCB in patients with CHA_2_DS_2_-VASc score of 2 (NCB 0.31; 95%CI -1.03 to 1.64). When we assigned WF of 1.5 and 2.0, there has been still positive and neutral NCB, respectively (Table 6; Fig. 3).

The NCB for DOACs was superior to warfarin regardless CHA_2_DS_2_-VASc score. When we assigned WF of 1.5 and 2.0, there has been still positive NCB. However, there was less NCB in patients taking DOACs compared to well-controlled warfarin (TTR ≥ 65%) when we assign WF of 1.0, 1.5 and 2.0 (Table 6; Fig. 3).

When AF patients were stratified according to sex, the superior NCB of any OACs versus no OACS was found in females with CHA_2_DS_2_-VASc score of 3 or more. Males with CHA_2_DS_2_-VASc score of 0–1 had a positive NCB for both warfarin and DOACs. Males with DOACs had a positive NCB in patients with CHA_2_DS_2_-VASc score of 0–2 while the NCB was negative in patients with CHA_2_DS_2_-VASc score of 3 or more. In the analysis of only well-controlled warfarin (TTR ≥ 65%) compared to no OACs, there was improved NCB in males with CHA_2_DS_2_-VASc score of 3 or more (NCB 0.43; 95%CI -0.16 to 1.01). In the analysis of DOACs compared to warfarin, there was positive NCB in females with CHA_2_DS_2_-VASc score of 3 or more and males with CHA_2_DS_2_-VASc score of 0–2. Compared to well-controlled warfarin (TTR ≥ 65%), there was less NCB in AF patients regardless sex and CHA_2_DS_2_-VASc score (Table 7).

**Table 7:** Net clinical benefit of all patients, male patients and female patients with atrial fibrillation taking oral anticoagulants based on CHA_2_DS_2_-VASc score

Antithrombotic strategy	NCB (overall)	NCB (male)	NCB (female)
Any OACs vs. No OACs			
CHA_2_DS_2_-VASc of 0–1	0.04 (0.04 to 0.05)	0.30 (0.19 to 0.41)	-1.55 (-3.16 to 0.07)
CHA_2_DS_2_-VASc of 2	-1.78 (-2.07 to -1.49)	-2.31 (-2.84 to -1.78)	-0.49 (-0.49 to -0.49)
CHA_2_DS_2_-VASc of ≥ 3	0.52 (0.33 to 0.73)	-2.06 (-2.36 to -1.75)	2.75 (2.09 to 3.43)
Warfarin vs. No OACs			
CHA_2_DS_2_-VASc of 0–1	-0.17 (-0.28 to -0.05)	0.14 (0.11 to 0.16)	-2.23 (-4.80 to 0.34)
CHA_2_DS_2_-VASc of 2	-1.97 (-2.29 to -1.65)	-2.60 (-3.15 to -2.05)	-0.36 (-0.50 to -0.23)
CHA_2_DS_2_-VASc of ≥ 3	0.38 (0.19 to 0.58)	-2.17 (-2.48 to -1.86)	2.61 (1.94 to 3.27)
DOACs vs. No OACs			
CHA_2_DS_2_-VASc of 0–1	1.18 (0.70 to 1.66)	1.23 (0.73 to 1.73)	0.91 (-0.88 to 2.71)
CHA_2_DS_2_-VASc of 2	0.31 (-1.03 to 1.64)	1.41 (1.03 to 1.79)	-1.53 (-6.08 to 3.01)
CHA_2_DS_2_-VASc of ≥ 3	2.19 (1.82 to 2.57)	-0.54 (-3.09 to 2.02)	4.38 (3.53 to 5.23)
Warfarin (TTR ≥ 65%) vs. No OACs			
CHA_2_DS_2_-VASc of 0–1	-0.33 (-1.31 to 0.64)	-0.54 (-1.73 to 0.65)	0.91 (-0.88 to 2.71)
CHA_2_DS_2_-VASc of 2	-0.63 (-1.12 to -0.13)	-2.25 (-3.05 to -1.44)	0.46 (1.40 to -0.49)
CHA_2_DS_2_-VASc of ≥ 3	2.63 (2.52 to 2.73)	0.43 (-0.16 to 1.01)	5.52 (4.83 to 6.22)
DOACs versus Warfarin			
CHA_2_DS_2_-VASc of 0–1	1.35 (0.80 to 1.90)	1.10 (0.67 to 1.53)	3.15 (-1.21 to 7.51)
CHA_2_DS_2_-VASc of 2	2.28 (0.96 to 3.60)	4.01 (3.61 to 4.40)	-1.17 (-5.81 to 3.48)
CHA_2_DS_2_-VASc of ≥ 3	1.81 (1.10 to 2.53)	1.63 (-1.52 to 4.79)	1.77 (1.29 to 2.26)
DOACs versus Warfarin (TTR ≥ 65%)			
CHA_2_DS_2_-VASc of 0–1	1.51 (-0.20 to 3.23)	1.77 (-0.23 to 3.77)	0.00 (0.00 to 0.00)
CHA_2_DS_2_-VASc of 2	0.93 (-0.12 to 1.98)	2.43 (1.87 to 2.99)	-1.99 (-5.88 to 1.91)
CHA_2_DS_2_-VASc of ≥ 3	-0.43 (-1.19 to 0.33)	-0.97 (-4.32 to 2.39)	-0.16 (-0.67 to 0.36)

NCB = net clinical benefit, OACs = oral anticoagulants, DOACs = direct oral anticoagulants, TTR = time in therapeutic range

## Discussion

To the best of our knowledge, there were several clinical trials demonstrating the OACs can reduce thromboembolic events in AF patients [2, 3]. However, there was increased MB rate including ICH from these medications. Previous trial by Singer et al. has shown that there was a positive NCB between TE rate and ICH in AF patients with CHADS_2_ of 2 or more among patients taking warfarin [14]. Thereafter, Olesen et al. showed that there was a positive NCB between TE rate and bleeding events in those patients with CHA_2_DS_2_-VASc score of 2 or more receiving warfarin [15].

This study was conducted in AF patients with OACs including warfarin and DOACs and stratified patients according to CHA_2_DS_2_-VASc score. The TE rate and the MB rate increased according to CHA_2_DS_2_-VASc score. The survival analysis illustrated that the OACs reduced TE rate while increased MB rate in those anticoagulated patients with CHA_2_DS_2_-VASc score of 2 or more with statistical significance.

Although most anticoagulated patients in this cohort study had a low bleeding risk of OACs reflecting from low HAS-BLED score (83.9%), there was significant increased MB rate in those patients with CHA_2_DS_2_-VASc score of 2 or more in patients receiving any OACs driven by the use of warfarin. Nevertheless, in the group of CHA_2_DS_2_-VASC score of 2 or more, patients treated with DOACs had higher MB rate than patients without OACs, though the difference was not statistically significant. The results of DOACs cohort were consistent with previous DOACs trials showing that these medications did not increase major bleeding events and reduce ICH [10–13].

When the NCB between TE and MB rate was analyzed, this cohort study showed that the positive NCB of any OACs in AF patients with CHA_2_DS_2_-VASc score of 3 or more while there was negative NCB of any OACs in those patients with CHA_2_DS_2_-VASc score of 2. Our results were not consistent with previous VKA trial from Olesen et al. However, previous aforementioned trial defined bleeding events including all bleedings in gastrointestinal tract, urinary tract, airways and ICH [15]. No major or minor bleeding events was classified in Olesen’s trial [15]. This led to the benefit of thromboembolic reduction outweighed the risk of bleeding events from inclusion of minor bleeding.

However, Singer et al. showed that there was the positive NCB between TE rate and ICH in those patients with CHADS_2_ score of 2 or more receiving warfarin [14]. Previous trials have demonstrated that the stroke risk in AF patients with CHADS_2_ score of 2 was 4.0% which was consistent with CHA_2_DS_2_-VASc score of 4 [20, 21]. For this evidence, those trial should reflect the positive NCB of warfarin in patients with CHA_2_DS_2_-VASc score of 4 as well. Nevertheless, those trial used only ICH for calculating NCB that was different from our trials.

When this trial classified patients according to the types of OACs, the NCB in patients with warfarin was similar to the results of any OACs cohort while there was positive NCB in patients with DOACs and CHA_2_DS_2_-VASc score of 0–1 and 3 more. This showed that the patients taking DOACs had lower MB rate compared with patients taking warfarin leading to more NCB as aforementioned results. This was confirmed by the NCB for DOACs was superior to warfarin regardless CHA_2_DS_2_-VASc score.

Previous trials demonstrated that Asian patients had more bleeding events compared with western patients [22–27]. This led to the negative NCB in those patients with warfarin in our cohort study. Because DOACs did not increase MB rate and decreased ICH, patients with DOACs and CHA_2_DS_2_-VASc score of 0–1 and 3 more had the positive NCB.

In addition, prior cohort study has shown the influence of sex was appeared to be associated with stroke and MB risk. Females have been associated with increased stroke risk while males have been associated with increased MB risk. The benefit of OACs favored in females [28]. This supported our study demonstrating that the NCB of OACs was more positive and negative according to increased CHA_2_DS_2_-VASc score in females and males, respectively.

However, our cohort study had several limitations. First, most patients in this trial were prescribed warfarin (91.1%) while patients without OACs may be prescribed other antithrombotic therapy such as antiplatelets. An ischemic stroke had many mechanisms and some patients might have large-artery atherosclerosis mechanism [29]. This mechanism of ischemic stroke was prevented by antiplatelets leading to decreased benefit of thromboembolic prevention in NCB formula. However, this study recruited only AF patients, so most TE rate in these patients was expected to be from thromboembolism and previous trial has shown that antiplatelet alone increased the risk of ischemic stroke/TIA with statistically significance [30]. Second, only 8.9% of OACs was DOACs leading to limit the power for interpretation of TE and MB rate. The lower prevalence of DOACs uses might cause the lower expected event rate and led to difficult for interpretation of NCB. However, this trial was the first study demonstrating the positive NCB in patients with DOACs. Finally, this study enrolled only Thai AF patients leading to limit the generalizability in other races.

## Conclusions

AF patients with CHA_2_DS_2_-VASc score of 3 or more regardless warfarin or DOACs had a positive NCB. The NCB of OACs was more positive for DOACs compared to warfarin and for females compared to males.

## Data Availability

The dataset that was used to support the conclusion of this study is included within the manuscript. Any other additional data will be made available upon request to the corresponding author.

## Abbreviations

AF: Atrial fibrillation
ATRIA: AnTicoagulation and Risk Factors In Atrial Fibrillation
CI: Confidence interval
CIEDs: Cardiovascular implantable electronic devices
CKD: Chronic kidney disease
CREC: Central Research Ethics Committee
CT: Computed tomography
DOACs: Direct oral anticoagulants
HR: Hazard ratio
ICH: Intracranial hemorrhage
ICH: GCP-International Conference on Harmonization for Good Clinical Practice Guidelines
LVEF: Left ventricular ejection fraction
MB: Major bleeding
MRI: Magnetic resonance imaging
NCB: Net clinical benefit
OACs: Oral anticoagulants
SD: Standard deviation
TE: Thromboembolic
TIA: Transient ischemic attack
TTR: Time in therapeutic range
WF: Weighting factor
